# Neutrophil Extracellular Traps (NETs) in Cancer Metastasis

**DOI:** 10.3390/cancers13236131

**Published:** 2021-12-06

**Authors:** Christof Kaltenmeier, Richard L. Simmons, Samer Tohme, Hamza O. Yazdani

**Affiliations:** Department of Surgery, University of Pittsburgh Medical Center, Pittsburgh, PA 15213, USA; kaltenmeierct2@upmc.edu (C.K.); simmonsrl@upmc.edu (R.L.S.); tohmest@upmc.edu (S.T.)

**Keywords:** neutrophil plasticity, neutrophil extracellular traps, tumor microenvironment, metastasis

## Abstract

**Simple Summary:**

Neutrophil Extracellular Traps (NETs) are formed following the activation of neutrophils and play an important role in the development of cancer, especially metastatic disease. In this review, we will portray the role of Neutrophils/NETs in the tumor microenvironment and circulation. We will furthermore discuss the role of neutrophil reverse migration, NET-mediated pre-metastatic niche formation, and possible treatment strategies to decrease metastatic cascade.

**Abstract:**

Metastasis is the leading cause of cancer related morbidity and mortality. The metastatic process involves several identifiable biological stages, including tumor cell dissemination, intravasation, and the extravasation of circulating cancer cells to facilitate colonization at a distant site. Immune cell infiltration and inflammation within the tumor microenvironment coincide with tumor progression and metastatic spread and are thought to be the key mediators of this complex process. Amongst many infiltrating cells, neutrophils have recently emerged as an important player in fueling tumor progression, both in animal models and cancer patients. The production of Neutrophil Extracellular Traps (NETs) is particularly important in the pathogenesis of the metastatic cascade. NETs are composed of web-like DNA structures with entangled proteins that are released in response to inflammatory cues in the environment. NETs play an important role in driving tumor progression both in experimental and clinical models. In this review, we aim to summarize the current advances in understanding the role of NETs in cancer, with a specific focus on their role in promoting premetastatic niche formation, interaction with circulating cancer cells, and in epithelial to mesenchymal transition during cancer metastasis. We will furthermore discuss the possible role and different treatment options for targeting NETs to prevent tumor progression.

## 1. Introduction

The term “metastasis” refers to the development of secondary tumor foci in a location other than the original or primary location. Cancer metastases remain the leading cause of cancer-related morbidity and mortality in patients and a major cause of treatment failure [1]. Despite its significance in cancer prognosis and management, little is known about the mechanistic pathways through which cancer cells spread. Several processes have been identified as playing important roles in metastasis formation: (1) the shedding of primary tumor cells, (2) the mechanism by which distant protumor niches develop, (3) tumor cell intravasation into and out of the circulation and lymphatic system, (4) how epithelial cancer cells acquire the motility and invasiveness characteristic of mesenchymal cells (epithelial to mesenchymal transition), (5) escaping host immunosurveillance, and (6) the development of a supportive tumor microenvironment [2,3]; all of these roles have been discussed over the past few decades, yet strategies to completely eradicate or prevent the development of metastases at an early stage have been unsuccessful. A further understanding of the mechanisms that are involved will be needed in order for effective therapeutic strategies to be devised.

The tumor microenvironment (TME) plays a crucial role in cancer metastasis and can significantly alter the therapeutic response and overall outcomes in patients [4]. Among other infiltrating immune cells (including myeloid-derived suppressor cells (MDSCs), dendritic cells, macrophages), neutrophils within the TME play a critical role in cancer progression and metastasis [5]. Neutrophils, originally known for their surveillance role, are among the early responders to infection and injury [6]. They are the most abundant circulating cells that are present in human blood and are required to initiate a series of defense mechanisms such as the phagocytosis, killing, and digestion of invading pathogens through the release of various granule components [7].

Based on a commonly held belief, circulating neutrophils are considered to be short-lived, with a lifespan of 7–10 h in both humans and mice, and are specialized to fulfill specific tasks following bone marrow extravasation. However, recent studies provide experimental evidence that neutrophils can survive for even longer (12 h to 5 days), especially in the TME [8,9,10]. In fact, it is reported that pro-inflammatory cytokines such as interferon gamma (IFN-γ) can prolong the survival of neutrophils and can further promote their recruitment to the TME, leading to a pro-tumorigenic environment [11].

Neutrophils harbor a wide set of tools that are both pro- and anti-inflammatory in nature [7,12]. Neutrophils that are infiltrating or that are present within the TME are referred to as tumor-associated neutrophils (TANs). TANs comprise a significant portion of a developing tumor and are usually in a protumor state. A high percentage of TANs has been shown to correlate with an increase in tumor metastasis, tumor grade, and tumor stage [13,14,15,16]. In addition, the neutrophil to lymphocyte ratio is currently being utilized as a predictor for overall mortality and disease-free survival. TANs, through their various mechanisms, have been found to promote tumor metastatic colonization, to facilitate pre-metastatic niche, and to induce an immunosuppressive TME [17]. TANs are found in the heterogenous population and can adapt to their surrounding environment. Nutrient deprivation and low oxygen tension are common features of the TME. Under such conditions, TANs can reprogram their metabolism to break down fatty acids or to utilize certain amino acids (glutamate, proline) to fuel their tumorigenic/pro-metastatic functions [18]. With a lack of nutrients in the TME majority of infiltrating immune cells, CD4+ and CD8+ T cells in particular are rendered exhausted and non-functional, which can contribute to tumor growth and proliferation [19].

Neutrophil Extracellular Traps (NETs) have recently been identified as a novel mechanism that is able to contribute to various steps in tumor metastasis and immune escape [20]. NETs, originally discovered by Volker Brinkmann and Arturo Zychlinsky in 2004, are web-like structures that were originally thought to entrap invading pathogens such as bacteria, fungus, protozoa, and viruses and are composed of DNA chromatin that has been extruded upon neutrophil activation and that is decorated with histones and antimicrobial proteins [21]. Note: the detailed neutrophil biology and the NETosis process has been well studied and described in several excellent review papers [22,23,24,25]. Recent studies have reported that NETs can not only trap and sequester circulating cancer cells but that they can also fuel their progression by directly interacting with cancer [26,27,28,29]. Due to their ability to entrap, NETs serve as an adhesion substrate for cancer cells in promoting tumor metastasis and can exert both pro and anti-tumor properties [30]. For instance, NET components such as myeloperoxidase have been shown to inhibit tumor growth and metastasis, especially in human lung cancer [31]. However, they have also been shown to promote cancer cell extravasation and metastasis by means of extracellular matrix degradation [32]. NETs are also involved in the epithelial to mesenchymal transition of cancer cells by regulating the multiple cellular pathways that further support tumor dissemination and metastatic spread. A recent study showed that NETs, when cocultured with gastric cancer cells, enhanced the expression of E-cadherin and vimentin and increased cancer cell migration [33]. In addition, elevated levels of NETs were also observed in patients with advanced gastric cancer and were found to be associated with increased metastasis.

Based on these findings, the primary focus of this review is to summarize the current advances in understanding the role of NETs in cancer progression through premetastatic niche establishment, direct cancer cell interaction, epithelial to mesenchymal transition, and facilitating cancer metastasis. In addition, strategies to prevent NET formation with different therapeutic options will be discussed.

## 2. Neutrophil Plasticity in the Tumor Microenvironment

**Figure 1:**
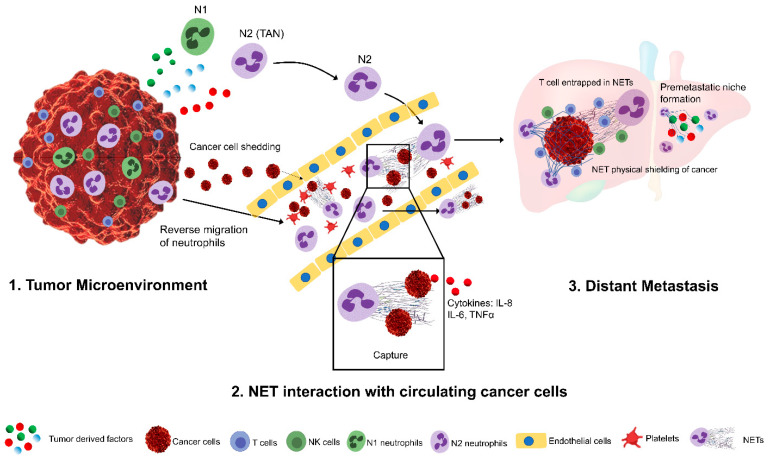
A simplified schematic representation of tumor-associated neutrophils (TANs) in promoting tumor metastasis. In the tumor microenvironment, tumor-derived factors can promote the recruitment, activation, and phenotypic differentiation of tumor-associated neutrophils (**1**). TANs can reverse migrate into the blood stream and can capture the circulating cancer cells, shedding from primary tumors through the induction of Neutrophil extracellular traps. NETs can further promote the invasion and migration of these captured tumor cells into the distant sites and shields them from cytotoxic lymphocytes (**2**). Tumor-derived factors further promote the development of pre-metastatic niches that facilities neutrophil infiltration and increased NET deposition (**3**).

It is well known that several cell types of immune cells, including the innate (neutrophils, macrophages, dendritic cells, NK cells) and adaptive (T cells and B cells) types, are present in the TME and play a key role in cancer biology [34]. Since neutrophils, among many other tumor infiltrating cells, make up a significant proportion of multiple types of cancer, their role has been controversial. Experimental evidence from numerous studies suggests that several tumor-derived factors promote the recruitment and activation of tumor-associated neutrophils (TANs) (Table 1). Once recruited, TANs can possess either antitumor properties by promoting tumor cytotoxicity or a tumor supporting role by stimulating tumor cell growth via invasion and migration (Table 2). Recent evidence suggests that TANs can retain functional plasticity and can be phenotypically altered when exposed to various TME signals such as transforming growth factor (TGF) -β or Type 1 interferon signals (Figure 1).

Neutrophils were long considered to be a homogeneous population that displayed important antimicrobial functions such as phagocytosis, enzymatic release through degranulation, and neutrophil extracellular trap (NETs) formation [82]. More recently, emerging evidence has shown that neutrophils are transcriptionally active cells that are often heterogeneous and that can be identified based on their density, cellular morphology, cell surface protein expression, enzymatic activity, and physical properties [7]. Since neutrophils are programmed to respond to numerous inflammatory stimuli that can shift their phenotype, Fridlender et al. in 2009 were among the first group to suggest a delineation between antitumorigenic and pro-tumorigenic tumor-infiltrating neutrophils, termed N1 and N2, respectively [83]. They discovered that TGF-β, an immunosuppressive cytokine that was overexpressed in murine lung tumor cells (AB12 and LRK) polarized neutrophils into a pro-tumorigenic phenotype (N2) and that inhibiting the TGF-β receptor on cancer cells led to an increase in neutrophil infiltration with an anti-tumorigenic phenotype (N1). Interestingly, they further showed that neutrophil depletion, following TGF-β blockade, significantly increased tumor growth, whereas in the control group, neutrophil depletion led to a decrease in tumor growth and volume. In the following study, the author further utilized state-of-the-art transcriptomic analysis to further delineate neutrophil plasticity within the TME [84]. Neutrophils isolated from the spleen and circulation from the AB12-injected tumor-bearing mice revealed significant transcriptional alteration that was characterized by excessive reactive oxygen species, nitric oxide production, and arginase expression.

Tumor-derived factors, aside from promoting a pro-tumorigenic shift in neutrophils, can also induce a phenotypic shift of infiltrating neutrophils toward an anti-tumor state [85]. At the developing stage of non-small-cell lung cancer (NSCLC), cytokines such as IFN-γ and GM-CSF are shown to drive the neutrophil differentiation towards expressing the co-stimulatory molecules CD13 (OX40) ligand, CD86, and 4-1BB ligand, specific neutrophil activation markers that promote anti-tumor activity [86]. Interestingly, a similar subset of these neutrophil populations was also observed in human head and neck cancer, especially those cancers that were associated with T cell activation [87].

The diversity of tumor myeloid-derived cells has been well documented in the literature; however, only a few studies have aimed to decipher the neutrophil subtypes that are involved in cancer. A single cell transcriptomic study of tumor-infiltrating cells from both murine (KP1.9) and human (NSLC) lung tumors revealed six and five distinctive sub-cell populations of TANs with a variety of phenotypic states when analyzed. Interestingly, further analysis showed that among these subsets, three common genomic pathway expressions were conserved between humans and mice, including (1) neutrophil expressing S100A8/9, ADAM8, and metalloproteinase (MMP) 9/8, (2) the expression of CCL3 and CSF-1, and (3) the significant expression of interferon regulatory factor (IRF) 7 and interferon-induced protein with tetratricopeptide repeats (IFIT) 1 respectively [88]. These genomic pathways outline the proinflammatory role of neutrophils in association with the increase in tumor growth, as both proteins such as S100A8/9 and cytokines CCL3 can be released from neutrophils through the induction of NETs and has been shown to recruit cancer cells, promote cancer progression, and facilitates metastatic niche formation [89,90].

## 3. Neutrophil Extracellular Traps in Cancer Metastasis

### 3.1. NETs and Premetastatic Niche Development

In addition to the pro- and anti-tumor promoting roles of neutrophils, recent studies have shown that neutrophils can reverse migrate and extravasate back into the bloodstream from inflamed and damaged tissue (Figure 1). This reverse migration was first explored in zebrafish and was explored more recently in murine models, suggesting a more complex neutrophil function within the TME [91,92,93]. A study from Wang et al. showed that in a liver sterile inflammation setting, murine neutrophils can exit the inflammatory environment and can further migrate to a secondary site, such as the lung and bone marrow, suggesting an important neutrophil role in developing an optimal environment (well characterized by Liu et al. review [94]) for cancer migration and metastatic spread [92]. It is well recognized that cancer cells are predisposed to metastasize to distant sites that harbor a favorable microenvironment that had been cultivated prior to the cancer cell arrival and colonization, as originally postulated by Paget’s “seed and soil” hypothesis [95]. There is compelling evidence that supports the hypothesis that neutrophils can be recruited to the site of pre-metastatic niches such as lung [96], liver [97], and omentum [98] and that they can aide in remodeling the local microenvironment through Neutrophil Extracellular Trap (NETs) and through the secretion of the pro-inflammatory mediators that further facilitate tumor cell extravasation and proliferation.

The role of NETs in the formation of cancer pre-metastatic niche and sustaining the secondary inflammatory environment is currently emerging. Since inflammation is the main contributor in the development of pre-metastatic niche, a recent finding observed by Yang et al. revealed a positive correlation between NET markers and the onset of colorectal cancer liver metastasis [55]. Through in vivo and in vitro studies, the authors revealed that tumor-derived factors from the primary tumor increased the frequency of neutrophil recruitment and excessive NET formation within the liver. In addition, the NETs were able to capture disseminated colorectal cancer cells (CRC), and furthermore, they subsequently induced the production of pro-inflammatory cytokines such as IL-8, IL-6, and TNF-α. This inflammatory cytokine storm further increased neutrophil recruitment and enhanced NET deposition, thus establishing a vicious cycle connecting NETs and the inflammatory niche in CRC liver metastasis [55].

In another study, the presence of NETs in the premetastatic omental niche correlated significantly in women with stage I and stage II ovarian cancer [98]. NETs were also detected in the omentum of tumor-bearing mice prior to metastasis. Utilizing genetically altered PAD4 KO mice, a key enzyme that regulates NETs formation, or treatment with DNAse in an orthotopic ovarian tumor model significantly reduced omental metastasis by 70%, thus indicating the early onset of tumor progression, especially for those with high metastatic potential, through the induction of NETs in the premetastatic omental niche, which, in turn, facilities distant tumor metastasis (Figure 1).

NET formation and the promotion of a pre-metastatic niche have been studied in several organ systems. Within the lung, neutrophils can awaken dormant cancer cells and can create a favorable milieu [99]. Environmental toxins such as cigarette smoking promote neutrophil activation and NET formation and subsequently tumor growth. Within the liver, Van der Windt et al. showed that non-alcoholic steatohepatitis (NASH) promotes NET formation [100]. In vitro, they showed that several free fatty acids can directly promote neutrophil activation. These findings provide another mechanism on how NETs are formed to promote the pre-metastatic niche.

To further support metastatic colocalization, the pre-metastatic niche may enhance angiogenesis and vascular permeability. Despite the lack of direct evidence of NETs in tumor angiogenesis and vascular permeability, a functional relationship between NETs and angiogenesis has been proposed in tissue regeneration as well as in vascular disease [20,101,102]. NET-embedded proteases such as neutrophil elastase (NE), myeloperoxidase (MPO), and matrix metalloproteases (MMP) are shown to cleave the vascular endothelial cadherin that compromises junction integrity, thus promoting vascular permeability [103]. In addition to vascular permeability, NETs have recently been shown to promote angiogenesis through the activation and clearance of senescent endothelial cells and through the further upregulation of proangiogenic factors such as VEGF [104].

### 3.2. NETs Promoting Epithelial to Mesenchymal Transition

The epithelial to mesenchymal transition (EMT) is a crucial mechanism that provides cancer cells with motility and invasiveness ability. Pieterse et al. were among the first groups to show that the coincubation of activated human neutrophils with endothelial cells resulted in vascular leakage and that they could promote EMT induction through the degradation of vascular endothelial cadherin (CD144) and the subsequent activation of Wnt/β-catenin signaling [105]. NET has also been shown to alter the typical epithelial morphology of human breast cancer cells (MCF7) into the mesenchymal phenotype by upregulating the expression of EMT transcriptional factors such as ZEB1 and snail (SNAI1) [106]. Similar observations were shown in the setting of human pancreatic cancer [107]. The coincubation of the activated NETs with human pancreatic cancer cell lines (BxPC3 and MIA PaCa2) significantly upregulated the mRNA levels of Snail, N-cadherin, and vimentin, which are associated with mesenchymal transition, and downregulated the expression of E-cadherin. Further analysis showed that the NET-activated EGFR/ERK signaling pathway in pancreatic cancer cells was crucial for EMT induction compared to other WNT, PI3K, and TGFβ signaling pathways. In addition, multiple clinical studies have shown a positive correlation of EMT activation with the presence of NETs in both primary and metastatic tumors. The potential of NETs to trigger EMT induction implies that targeting NETs during the initial stages of disease progression may provide beneficial outcomes.

Several NET components such as neutrophil elastase (NE), histones, and HMGB1 have been linked to the induction of cancer EMT. However, targeting NETs alone might not produce sufficient EMT suppression. Martins-Cordoso et al. showed that the treatment of NETs with DNAse, which effectively degrades DNA integrity, had a minor impact on the migratory effect of human breast cancer cells [106]. Similarly, a study published by Kajioka et al. showed that NET degradation did not halt the invasion and migration of primary pancreatic cancer metastasis to the liver, whereas treatment with the neutrophil elastase inhibitor (NEi) or recombinant human thrombomodulin (TM), which degrades NET-derived HMGB1, abrogated EMT induction [108]. Indeed, the degradation of NETs through DNAse treatment has shown minor effects on cancer EMT induction but has been found to attenuate the other steps that are involved in the metastatic cascade. It is possible that NET treatment with DNAse alone alters the effect of NET-bound proteins but not those that are released in a NET-independent fashion upon neutrophil activation, such as HMBG1. Martins-Cordoso et al. proposed that DNA integrity is dispensable in the need for cancer cell migration. In addition, treatment with TM not only prevented NET induction but also degraded HMGB1 protein.

### 3.3. Interaction between NETs and Circulating Cancer Cells

Tumor recurrence and the development of distant metastatic disease after curative intent resection remains an important and frequently encountered clinical problem. During surgery, the tumor tissue undergoes unavoidable damage that can lead to the shedding of tumor cells into the bloodstream and lymphatic circulation [109]. Furthermore, surgical stress leads to a surge of inflammatory cytokines and the increase of innate immune cells, both in the tissue and in circulation. Studies have shown that following resection, especially in patients with hepatocellular carcinoma, the number of circulating tumor cells (CTC) can dramatically increase in the post-operative period [110]. CTCs can then adhere to the endothelium, infiltrate at distant sites, and continue to proliferate. It is therefore not surprising that the CTCs that are found within the blood post-resection have been linked to the development of regional and metastatic disease and are prognostic factors for decreased overall patient survival [111]. A wide array of studies has shown that the survival of CTCs and adhesion at distant sites following surgery can be linked to systemic inflammation, especially the presence of neutrophils and NETs. Najmeh et al. have shown that during systemic inflammation, NETs can entrap human (AF49) and mouse (H59) lung carcinoma circulating tumor cells [27]. Their study showed that within the post-operative inflammatory period, β1-integrin expression was upregulated in AF49 lung carcinoma cells and promoted the adhesion of CTCs to NETs in different models. Furthermore, they showed that the cancer cells trapped within NET chromatin led to higher numbers of hepatic metastatic disease. The destruction of NET chromatin using DNAse abrogated cancer cell adhesion to NETs and diminished metastatic disease. In addition, NET chromatin is embedded with a variety of proinflammatory molecules that are crucial to the capture of tumor cells and alterations in the endothelial cells to augment adhesion and migration. Specifically, the roles of VCAM-1 and ICAM-1 in mediating the interaction between neutrophils and CTCs as well as CTCs and endothelial cells have been studied [112].

Several studies have also investigated the interaction of NETs in promoting platelet aggregation and clot formation, thereby promoting cancer progression and invasion [113,114]. Platelet interaction with activated neutrophils is a potent NET inducer [115]. In addition, NETs can amplify platelet activation, aggregation, and thrombin activation. CD62P that is expressed on activated platelets can bind to P-selectin glycoprotein ligand-1 (PSGL-1) on the surface of neutrophils and can thereby lead to enhanced intracellular signaling cascades and the enhanced expression of additional adhesion molecules on the surface of the platelets [116]. The uncontrolled activation of platelets thereby leads to blood vessel occlusion and the entrapment of more NETs and circulating cancer cells.

### 3.4. Role of NETs in the Growth of Micrometastatic Disease

Circulating cancer cells that invade distant sites often find themselves in a hostile microenvironment lacking adequate nutrition, angiogenic, and pro-inflammatory factors. Cells then undergo a state of dormancy, which is often one of the reasons why patients present with metastases long after treatment and after the removal of the primary tumor. It is still not fully understood what triggers a change in the balance between the signals that keep disseminated cancer cells under growth arrest or that promote the development of metastases. However, localized inflammation has been linked to the awakening of these dormant cells. Albruenges et al. found that experimental pulmonary inflammation following tobacco smoke exposure in LPS led to NET formation and the awakening and proliferation of prior dormant breast (MCF-7) cancer cells [99]. This effect was mediated by NETs through the cleavage of integrins in the extracellular matrix by neutrophil elastase (NE) and Matrix metalloproteinase-9 (MMP-9). This subsequently led to proliferation via the activation of integrin alpha-3beta-1 signaling.

The presence of NETs in the TME has also been linked to alterations in the bioenergetic profile of these cancer cells. NET-rich TME cancer cells often favor energy production via mitochondrial respiration and oxidative phosphorylation in order to position themselves in a hostile environment. In both in vivo and in vitro experiments, we have previously shown that cancer cells undergo mitochondrial biogenesis along with mitochondrial fusion and fission in the presence of NETs [29]. Our group found that NETs mediate this effect through peroxisome proliferator-activated receptor γ coactivator 1-α (PGC1α), thereby leading to an energy boost to promote anabolic growth. This effect was further mediated by NE through Toll-like receptor (TLR) 4 signaling. In addition, with ongoing tumor growth, more neutrophils infiltrate the TME and form NETs, leading to increased cancer cell proliferation and an ongoing vicious cycle. The number of chemokine receptors, such as CXCL1 and CXCL2 are expressed on neutrophils following surgical stress; however, they can also be found on some tumor cells [117]. These chemokines, in addition to a number of inflammatory mediators, can trigger neutrophil migration and NETosis. Within the TME, NETs have been shown to coat and shield tumor cells against infiltrating CD8+ and NK cells (Figure 1) [117]. Teijeira hypothesized that this protective mechanism leads to armored tumor cells, resulting in a loss of protection when DNAse is used. Decreased tumor cytotoxicity is mediated through direct contact inhibition and the physical separation of malignant cells from immune cells [117]. In addition, their findings also suggest that lymphocyte migration could be diminished upon NET exposure due to functional impairment. A study by Sivanandham et al. has proven that T cells can become trapped within NETs and thereby promote apoptosis, leading to decreased T cell numbers in an SIV-model [118].

## 4. NETs as a Potential Therapeutic Target

NETs harbor a variety of different proteins within their structure and thereby gain the ability to foster metastatic dissemination and evasion from the immune system. Given this knowledge, targeting the formation or activity of NETs could potentially play an important role in cancer treatment. At this moment, the majority of therapeutics targeting NETs either lead to the disruption of the chromatin or inhibit the formation of NETs through pathway inhibition.

Protein arginine deiminase 4 (PAD4) plays a crucial role in the formation of NETs via histone citrullination to promote chromatin decondensation and the expulsion of chromosomal DNA [119]. Global PAD4 knockout mice have shown diminished NET production and decreased primary and metastatic cancer growth compared to wild-type mice [26,27,28,29]. Similar effects of decreased micrometastatic disease have been shown upon the targeting neutrophil elastase with GW311616A or when using an MPO-inhibitor in mice [29].

The destruction of NETs as the primary target within tumors may represent another effective approach. DNA, the core structure of NETs, can be targeted using DNAse I. In, fact DNAse I treatment has shown promising results in preclinical murine models as well as clinical trials in patients with lupus nephritis through intravenous injection or cystic fibrosis administered by inhalation [120,121]. Teijeira et al. has shown that in vivo DNAse treatment can abrogate the protective effect of NETs on cancer cells and can render them susceptible to cytotoxic cells. The intravenous administration of DNAse-I however requires multiple injections due to the short half-life in circulation [122]. There are only a few clinical trials that have tested the efficacy of recombinant DNAse on cancer patients, including head and neck cancer (NCT00536952) and myeloid leukemia (NCT02462265) trials, that have shown antitumor activity along with the safety of these patients.

Furthermore, blocking the direct crosstalk of NETs and cancer cells has been shown to be promising in abrogating the NET effect on cancer cells. As previously described, NETs can have a wide variety of effects on cancer cells, including alterations in metabolism, invasion, cell cycle progression, and metastatic growth. Several mediators of cancer–NET interaction have been identified, including TLR4–NE, tumor specific integrins (α5β1 and ανβ3), and most recently, a surface protein called CCDC25 [117]. Targeting these receptors using specific antagonists or knockouts has shown promising results in decreasing tumor cell migration, metastatic niche formation, and growth.

## 5. Conclusions

In recent years, significant advances have been made to understand the effect and mechanism of neutrophils and NETs in aiding tumor growth, progression, and metastasis. Neutrophils display a large amount of plasticity within the TME and pose a major challenge in the therapeutic targeting of cancer and metastasis. Different types of tumors and disease stages generate various cancer-derived factors that can promote the phenotypic shift of neutrophils towards a pro- or antitumor type. The identification of specific markers and the better characterization of these two populations could improve the specific targeting of pro-tumor neutrophils. In addition, understanding the biology of how these different neutrophil populations predispose NETs along with the type of traits can better elucidate the downstream effects of NETs in tumor growth and progression. On the other hand, NETs are frequently found within the TME and can promote pro-tumorigenic effects such as increased cancer metabolism, invasion, and growth; however, they can also directly interact with infiltrating immune cells to protect the tumor. Targeting NETs, specifically those that are formed from the protumor neutrophil subgroup, could play a promising role in anticancer therapy; however, due to the complexity and multifaced roles of neutrophils within the TME, further investigation is needed.

## Figures and Tables

**Table 1:** Tumor-derived factors in recruiting neutrophils and stimulating Neutrophil Extracellular Traps (NETs) formation.

Tumor-Derived Factor	Neutrophil Receptor	Neutrophil Response/Action	Cancer Type	References
CXCL1/KC	CXCR2	TAN Chemotaxis, promote metastasis, chemoresistance	Breast cancer, Colorectal cancer, Lung cancer, Melanoma, Pancreatic cancer	[35,36,37,38,39]
CXCL2/MIP2α	CXCR1, CXCR2	TAN Chemotaxis, promote metastasis, chemoresistance	Breast cancer, HCC, Lung cancer, Melanoma, Pancreatic cancer	[35,36,38,39,40,41]
CXCL5	CXCR2	TAN Chemotaxis, EMT induction	Breast cancer, Colorectal cancer, HCC, Lung cancer, Pancreatic cancer	[35,36,38,41,42,43]
CXCL6/GCP-2	CXCR1, CXCR2	TAN Chemotaxis,	Melanoma	[35,44]
CXCL12	CXCR4		Ovarian cancer	[45,46]
HMGB1	TLR4, RAGE	TAN chemotaxis, NET formation, tumor angiogenesis and metastasis	Colorectal cancer, HCC	[47]
GM-CSF	GM-CSFR	TAN chemotaxis, promote tumor angiogenesis and metastasis	Colorectal cancer, Pancreatic cancer, Lung cancer, Thyroid cancer	[48,49,50,51]
GC-SF	GC-SFR	TAN chemotaxis, promote tumor growth and metastasis	Breast cancer, Pancreatic cancer, Lung cancer	[50,52,53,54]
IL-8	CXCR1, CXCR2	TAN Chemotaxis and activation	Colorectal cancer, HCC, Ovarian cancer, Melanoma, Pancreatic cancer	[40,50,55,56,57]
IL-17	IL-17R	TAN Chemotaxis, activation, tumor growth and metastasis	Breast cancer, Colorectal cancer	[49,58,59]
IL-33	ST2	TAN Chemotaxis and activation	Colorectal cancer	[60,61]
IL-1β		Tumorigenesis and metastasis	Colorectal cancer	[62]
TNF-α	TNF-αR	TAN Chemotaxis	Colorectal cancer	[63]
TGF-β	TGF-βR	Promote tumor growth	Breast cancer, Colorectal cancer	[64,65]
LTB4	BLT1	TAN chemotaxis, promote tumor growth	Lung Cancer, Pancreatic cancerHuman pancreatic cancer/LKR13 (lung tumor model)	[66,67]
Cathepsin C	PR3	TAN Chemotaxis and NET formation	Breast Cancer	[68]

**Legend**: HCC: hepatocellular carcinoma; CXCL: C-X-C motif chemokine ligand; CXCR: chemokine receptor; MIP2α: macrophage inflammatory protein-2 alpha; GCP-2: granulocyte chemotactic protein-2; IL: interleukin; HMGB1: high-mobility group box 1; TLR: toll-like receptor; RAGE: receptor for advanced glycation endproducts; GM-CSF: granulocyte–macrophage colony-stimulating factor; GM-CSFR: granulocyte–macrophage colony-stimulating factor receptor; GC-SF: granulocyte colony-stimulating factor; TNF-α: tumor necrosis factor-alpha; TNF-αR: tumor necrosis factor-alpha receptor; LTB4: leukotriene B4.

**Table 2:** Pro and anti-tumor properties of neutrophils and their role in tumor process.

Phenotype	Neutrophil Component	Tumor Process	References
N2 (Pro-tumor)	MMP9, VEGF, BV8	Angiogenesis	[44,69,70]
	ARG1, INOS, CCL17	Suppression of immune cells	[71,72]
	HGF, OSM	Cancer metastasis	[73,74]
	NE, PGE2, NETs	Cancer growth	[75,76]
	ROS, H_2_O_2_	Carcinogenesis	[72]
N1 (anti-tumor)	ROS, HOCL	Tumor cell lysis	[77]
	TRAIL	Tumor cell apoptosis	[78]
	ADCC	Tumor cell cytotoxicity	[79]
	TNFα, NE, IFN-γ	Promote T cell proliferation	[80]
	Cathepsin G	H_2_O_2_ dependent tumor cell cytotoxicity	[81]

**Legend:** MMP: matrix metalloproteinase; VEGF: vascular endothelial growth factor; ARG1: arginase 1; INOS: inducible nitric oxide synthases; CCL7: C-C motif chemokine ligand 7; HGF: hepatocyte growth factor; OSM: oncostatin M; NE: neutrophil elastase; PGE: prostaglandin E2; ROS: reactive oxygen species; H_2_O_2_: hydrogen peroxide; HOCL: hypochlorous acid; TRAIL: tumor necrosis factor-related apoptosis-inducing ligand; ADCC: antibody dependent cellular cytotoxicity.

## Data Availability

Not applicable.
